# The Actin-Binding Proteins Eps8 and Gelsolin Have Complementary Roles in Regulating the Growth and Stability of Mechanosensory Hair Bundles of Mammalian Cochlear Outer Hair Cells

**DOI:** 10.1371/journal.pone.0087331

**Published:** 2014-01-27

**Authors:** Jennifer Olt, Philomena Mburu, Stuart L. Johnson, Andy Parker, Stephanie Kuhn, Mike Bowl, Walter Marcotti, Steve D. M. Brown

**Affiliations:** 1 Department of Biomedical Science, University of Sheffield, Sheffield, United Kingdom; 2 Medical Research Council (MRC), Mammalian Genetics Unit, Harwell, United Kingdom; University of Minnesota, United States of America

## Abstract

Sound transduction depends upon mechanosensitive channels localized on the hair-like bundles that project from the apical surface of cochlear hair cells. Hair bundles show a stair-case structure composed of rows of stereocilia, and each stereocilium contains a core of tightly-packed and uniformly-polarized actin filaments. The growth and maintenance of the stereociliary actin core are dynamically regulated. Recently, it was shown that the actin-binding protein gelsolin is expressed in the stereocilia of outer hair cells (OHCs) and in its absence they become long and straggly. Gelsolin is part of a whirlin scaffolding protein complex at the stereocilia tip, which has been shown to interact with other actin regulatory molecules such as Eps8. Here we investigated the physiological effects associated with the absence of gelsolin and its possible overlapping role with Eps8. We found that, in contrast to Eps8, gelsolin does not affect mechanoelectrical transduction during immature stages of development. Moreover, OHCs from gelsolin knockout mice were able to mature into fully functional sensory receptors as judged by the normal resting membrane potential and basolateral membrane currents. Mechanoelectrical transducer current in gelsolin-Eps8 double knockout mice showed a profile similar to that observed in the single mutants for Eps8. We propose that gelsolin has a non-overlapping role with Eps8. While Eps8 is mainly involved in the initial growth of stereocilia in both inner hair cells (IHCs) and OHCs, gelsolin is required for the maintenance of mature hair bundles of low-frequency OHCs after the onset of hearing.

## Introduction

The perception of hearing depends on the transduction of sound stimuli into electrical signals that are transmitted to the auditory afferent neurons. Crucial to this process is the opening of mechanically gated channels localized near the tips of stereocilia that protrude from the apical surfaces of hair cells to form hair-like bundles **[1]**, **[2]**. Stereocilia are finger-like projections with a core composed of an array of parallel, uniformly polarized actin filaments that are coupled to one another by several types of extracellular links **[3]**–**[5]**. The length of each stereocilium is scaled precisely to form the staircase-like structure of each hair bundle, the overall size and shape of which depends on location along the cochlea **[6]**. The development of this precise array of stereocilia mainly occurs during early postnatal stages through a process of elongation and thickening, as well as the elimination of redundant stereocilia **[5]**, **[6]**.

In the adult cochlea, stereociliary maintenance involves several actin-binding proteins such as espin **[7]**, **[8]**, twinfilin 2 **[9]**, gelsolin [**10**],the epidermal growth factor receptor substrate 8 (Esp8 [**11]**, **[12]**), Eps8-L2 [**13**], scaffolding proteins such as whirlin [**14]**, **[15]** and unconventional myosin motors including myosin XVa [**15**] and myosin IIIa [**16**]). Despite recent progress, we do not have a complete understanding of how most of these molecules are able to control the growth and maintenance of stereociliary bundles in hair cells. Gelsolinis an actin-capping and severing protein expressed in the shorter stereocilia of cochlear outer hair cells (OHCs) from aboutP0 to P15 [**10**]. In the absence of gelsolin, stereocilia appear to grow normally but by the onset of hearing (P12) they become long and straggly, indicating that gelsolin is involved in the actin regulation of stereocilia elongation [**10**]. Whirlin appears to act as a scaffold to other actin regulatory molecules such as the MAGUK protein p55 [**10**] and Eps8 [**11**] and gelsolin has recently been shown to interact with p55 [**10**].

Given that gelsolin and Eps8 are expressed in the stereocilia of OHCs, we hypothesized that they may co-operate during the growth and/or maintenance of the hair bundles. To test this hypothesis we undertook a morphological and physiological investigation of cochlear OHCs in knockout mice. We report that these actin regulatory proteins have a non-overlapping role in stereocilia elongation and while Eps8 is required for the initial elongation of the stereociliary bundle, gelsolin is crucial for maintaining the cohesion of the hair bundle structure after the onset of hearing.

## Results

### Hair cell transducer current in gelsolin knockout mice (*Gsn^tm1Djk^/Gsn^tm1Djk^*)

**Figure 1:**
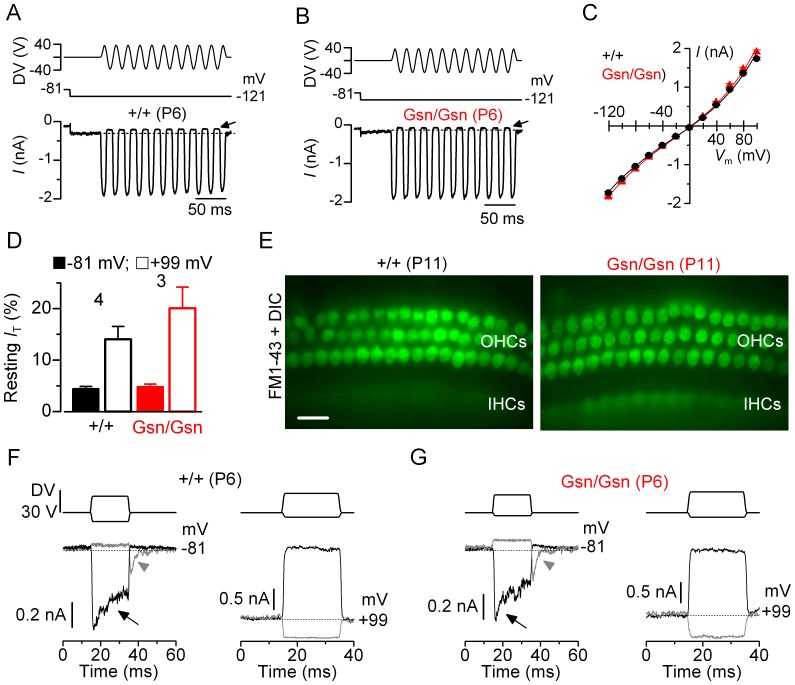
Mechanotransducer currents in outer hair cells from gelsolin knockout mice (*Gsn^tm1Djk^/Gsn^tm1Djk^*). A and **B**, Saturating mechanotransducer (MET) currents recorded from a control (**A**) and a gelsolin knockout (**B**) P6 apical-coil OHC. MET currents (bottom panels) were elicited by applying sinusoidal force stimuli to the hair bundles while changing the membrane potential between −121 mV and +99 mV in 20 mV nominal increments from the holding potential of −81 mV (middle panel). For clarity only responses at −121 mV are shown. The driver voltage (DV) signal of ±40 V at 50 Hz to the fluid jet is shown above the traces (negative deflections of the DV are inhibitory). The arrows indicate the closure of the transducer channels, i.e. disappearance of the resting current, during inhibitory bundle displacements. Dashed lines indicate the holding or resting current. **C**, Peak-to-peak current-voltage curves were obtained from four control and three knockout OHCs (P6) using 1.3 mM extracellular Ca^2+^. The fits through the data are according to eqn.1 (see Methods) with values: control *k* = 494±51, *V*_r_ = 2.3±0.4 mV, *V*_s_ = 40±3 mV, and γ = 0.41±0.01; gelsolin knockout *k* = 537±40, *V*_r_ = 1.6±0.3 mV, *V*_s_ = 40±2 mV, and γ = 0.42±0.01. **D**, Changes in the resting transducer current at −81 mV and +99 mV in control and knockout OHCs. The resting current is given by the holding current minus the current present during inhibitory bundle deflection. **E**, Fluorescence images with the DIC image superimposed from the control and knockout P11 cochleae taken after exposure to FM1-43 (both OHCs and IHCs were labelled by the dye). Scale bars: 20 µm. **F** and **G**, Driver voltages to the fluid jet (top) and transducer currents recorded at –81 mV (left panels) and +99 mV (right) from a control and a knockout gelsolin OHC respectively. At –81 mV, positive driver voltages (excitatory direction) elicited inward transducer currents that declined or adapted over time in control and knockout OHCs (arrows). A small transducer current was present at rest (dashed line) and inhibitory bundle displacements turned this off. Upon termination of the inhibitory stimulus, the transducer current in control and knockout OHCs showed evidence of rebound adaptation (arrowheads). These manifestations of MET current adaptation were absent at positive membrane potentials (e.g. +99 mV) and the resting current increased.

Given the location of gelsolin at the stereociliary bundles [**10**] we investigated its possible role in mechanotransduction. Mechanoelectrical transducer (MET) currents were recorded from postnatal day 6 (P6) OHCs by displacing their hair bundles in the excitatory and inhibitory direction using a piezo-driven fluid-jet (50 Hz sinusoidal force stimulus [**17]**, **[18]**). Upon moving the bundles in the excitatory direction (i.e. towards the taller stereocilia) and at negative membrane potentials, an inward MET current could be elicited in OHCs from both control (+/+) and knockout mice (**Figure 1A and B**). The maximal MET current in control OHCs (−1727±24 pA at −121 mV, *n* = 4) was not significantly different to that recorded in knockout (−1831±25 pA *n* = 3) OHCs. Any resting current flowing through open MET channels in the absence of mechanical stimulation was reduced when bundles were moved in the inhibitory direction (i.e. away from the taller stereocilia) in all control and knockout OHCs (**Figure 1A and B**, arrows). **Figure 1C** shows the size of the MET current at the different membrane potentials tested (nominally from −120 mV to +100 mV). Because the MET current reverses near 0 mV, it became outward when excitatory bundle stimulation was applied during voltage steps positive to its reversal potential (**Figure 1C**). The open probability of MET channels at rest near −81 mV and in 1.3 mM Ca^2+^ was also similar between control and knockout OHCs (**Figure 1D**). Note that the reduced driving force for Ca^2+^ influx at depolarized potentials (e.g. +99 mV in **Figure 1C**) caused the resting current to increase in both genotypes, suggesting a similar Ca^2+^ sensitivity of the transducer apparatus [**17]**, **[19]**.

The above experiments were performed on young animals (P6) since this age is the most reliable for recording accurate transduction currents from mouse hair cells [**20**]. We tested whether transduction was likely to be functional in more mature P10-P11 OHCs by using the styryl dye FM1-43 (**Figure 1E**), which is a permeant blocker of the hair cell transducer channel and used to assess the presence of the resting transducer current in hair cells [**21**]. Bath application of FM1-43 resulted in the selective labelling of hair cells from both genotypes with FM1-43, indicating the presence of a normal transduction process (**Figure 1E**).

**Figure 2:**
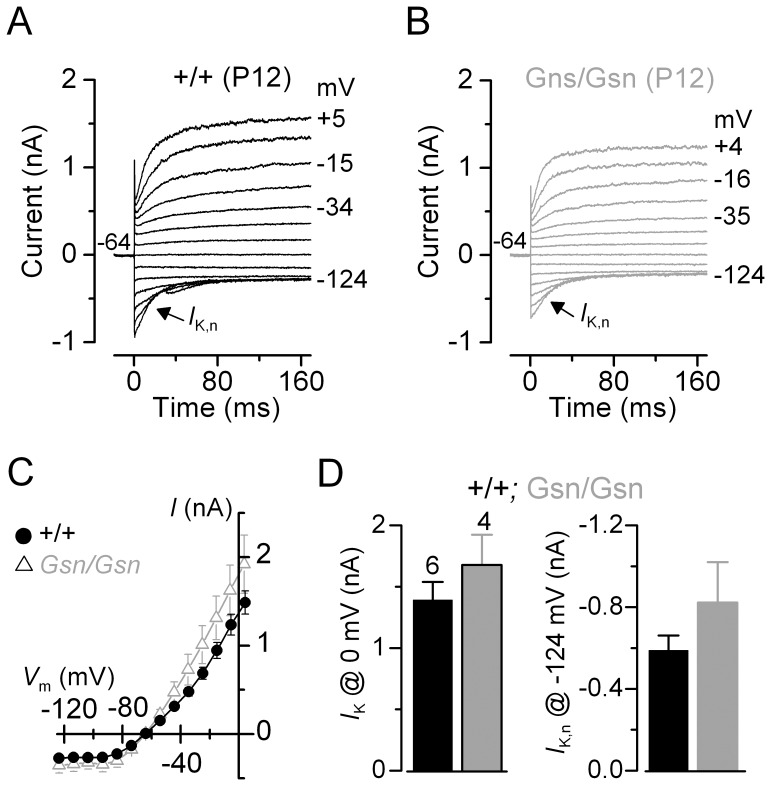
Gelsolin is not involved in the development of OHC basolateral currents in knockout mice (*Gsn^tm1Djk^/Gsn^tm1Djk^*). A and **B**, K^+^ currents recorded from mature control and knockout OHCs, respectively, elicited by depolarizing voltage steps (10 mV nominal increments) from –124 mV to more depolarized values from the holding potential of –64 mV. The K^+^ current characteristic of adult OHCs, *I*_K,n_, was similarly expressed in OHCs from control and knockout gelsolin mice. **C**, Steady-state current-voltage curves for the total K^+^ current in control (*n* = 6) and gelsolin knockout (*n* = 4) OHCs. **D**, Size of the total outward K^+^ current measured at 0 mV (left) and the isolated adult-type current *I*_K,n_, which was measured as the deactivating tail currents (difference between instantaneous and steady-state inward currents) at –124 mV [**24**].

We then investigated whether the absence of gelsolin affected the adaptation properties of the MET current by stimulating the hair bundles of OHCs using alternating excitatory and inhibitory mechanical step stimuli instead of sinusoids. In control P6 OHCs, excitatory bundle movements with non-saturating stimuli elicited rapid inward currents at a holding potential of –84 mV that declined or adapted over time (**Figure 1F**: arrow in left panel). Inhibitory hair bundle stimulation shut off the small fraction of the current flowing at rest (see also **Figure 1A**) and at the offset of large inhibitory steps, a transient rebound (downward dip: **Figure 2A**: arrowhead in left panel) was observed. Similar results were also obtained in the absence of gelsolin (**Figure 1G**, left panel). All these manifestations of MET current adaptation were absent when stepping the membrane potential to positive values (see also **Figure 1F and G**, right panels), in agreement with previous observations in hair cells from lower vertebrates and mice [**13]**, [19], **[22]**.

### OHC basolateral membrane properties develop normally in gelsolin knockout mice

Previous findings have shown that an absence of stereociliary proteins can cause a failure in the functional maturation of cochlear hair cells (e.g. Myo VI [**23**]; Eps8 [**12**]). Therefore, we investigated whether the absence of gelsolin affected the development of the OHC basolateral membrane currents. Potassium currents were elicited by applying a series of hyperpolarizing and depolarizing voltage steps in 10 mV nominal increments from the holding potential of –64 mV. We found that control and gelsolin knockout OHCs (P12) expressed the delayed rectifier K^+^ current *I*_K,n_ (**Figure 2A and B**), which is the major current component in adult mouse OHCs that appears from P8 onwards [**24**]. The amplitude of the total K^+^ current (**Figure 2C**) measured at 0 mV (**Figure 2D**, left) and the isolated *I*_K,n_ measured at –124 mV (**Figure 2D**, right) were similar between the two genotypes.

### Localisation of Eps8 in cochlear hair cells of gelsolin knockout mice (*Gsn^tm1Djk^/Gsn^tm1Djk^*)

**Figure 3:**
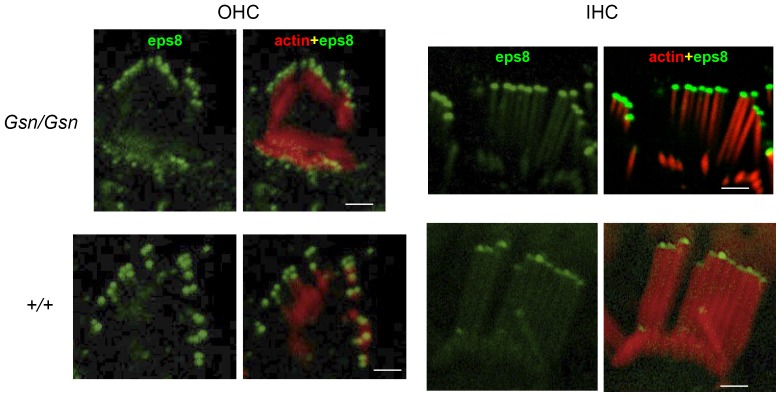
Eps8 localization in cochlear hair cells of gelsolin knockout mice (*Gsn^tm1Djk^/Gsn^tm1Djk^*). Cochlear whole mounts from 3 month old gelsolin knockout mice were stained with antibody to Eps8 (green) and phalloidin (red) to detect actin. Right hand panels show merge of Eps8 and actin. Eps8 localizes at the tip of stereocilia in both inner (IHC) and outer (OHC) hair cells. In IHC stereocilia Eps8 is present in the tips of both the taller and shorter rows. There was no obvious difference in localization pattern between wild type and gelsolin knockout mice. Scale bars: 10 µm.

Both Eps8 and gelsolin have been shown to be part of the whirlin complex in which whirlin appears to act as a scaffold to the other actin regulatory molecules which also includes proteins such as the MAGUK protein p55 and myosin XVa [**10]**, [15], **[25]**. We therefore undertook localization studies of Eps8 in the gelsolin knockout mouse. Confocal studies on the gelsolin knockout using an Eps8 antibody [**12**] showed that the protein was localized at the tips of stereocilia in both OHCs and inner hair cells (IHCs). We found no obvious difference in the localization pattern of Eps8 protein between wild type and gelsolin knockout mice (**Figure 3**).

### Localisation of gelsolin in cochlear hair cells of Eps8 knockout mice (*Eps8^tm1Ppdf^/Eps8^tm1Ppdf^*)

**Figure 4:**
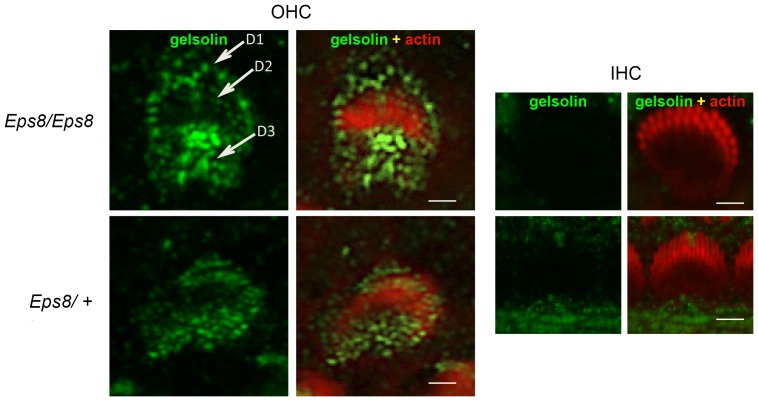
Gelsolin localization in cochlear hair cells of Eps8 knockout mice (*Eps8^tm1Ppdf^/Eps8^tm1Ppdf^*). Cochlear whole mounts from P8 mice were stained with antibody to gelsolin (green) and phalloidin (red) to detect actin. Right hand panels for OHC and IHC cells show merge of gelsolin and actin. Note the absence of IHC labeling. Gelsolin localizes to stereocilia of OHCs (domain D2) and is also detected outside of the stereocilia bundle in strial (D1) and neural (D3) domains of the apical hair cell surface. Gelsolin localization was not affected in the Eps8 knockout mice. Similar results were seen at P6 (data not shown). Scale bars: 10 µm.

We also undertook localisation studies of gelsolin in the Eps8 knockout mouse. Confocal studies using a gelsolin antibody [**10**] localized the protein to the stereocilia bundle of OHCs along the entire length of the cochlear duct. Gelsolin was confined to the OHCs and, like p55 [**25**], was not expressed in IHCs. Gelsolin is seen in OHCs from P0 till after P8 whereupon expression fades out by P15 [**25**]. Gelsolin is localized to the stereocilia of OHCs (domain, D2; see **Figure 4**) and outside of the stereocilia bundle in the strial (D1) and neural (D3) domains of the hair cells apical surface. Gelsolin was not detected in the stereocilia of IHCs [**10**]. We found that gelsolin localisation was not affected at P8 in Eps8 knockout mice (**Figure 4**). Similar results were seen at P6 (data not shown).

### Analysis of Eps8-gelsolin double heterozygous mice (*Eps8^tm1Ppdf^/+; Gsn^tm1Djk^/+*)

**Figure 5:**
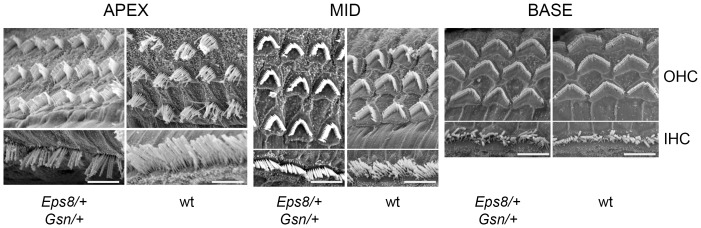
Ultrastructural analysis of cochlear hair cell stereocilia in Eps8-gelsolin double heterozygous mutant mice (*Eps^8tm1Ppdf^/+; Gsn^tm1Djk^/+*). SEM analysis of hair cell stereocilia bundles in Eps8-gelsolin double heterozygous mice and wild type litter mate controls at P27. OHCs and IHCs at the apex, mid and basal turns of the cochlea from Eps8-gelsolin double heterozygous mice showed similar hair bundles to those in wild type litter mate controls. Scale bars: 10 µm.

We carried out SEM analysis of stereocilia bundles along the length of the cochlear duct of Eps8-gelsolin double heterozygous adult mice and compared them to those in wild type littermate controls. The morphology of both OHC and IHC bundles appeared normal along the length of the cochlear duct in the double heterozygous (**Figure 5**). Neither the abnormal stereocilia phenotype seen in the apical turn of the cochlear duct of gelsolin homozygous mice [**10**] nor the shortened stereocilia seen in Eps8 homozygous mice [**12**] were seen in the double heterozygous mutant mice.

### Hair cell transducer currents in Eps8-gelsolin double homozygous mutant (*Eps8^tm1Ppdf^/Eps8^tm1Ppdf^; Gsn^tm1Djk^/Gsn^tm1Djk^*) mice

**Figure 6:**
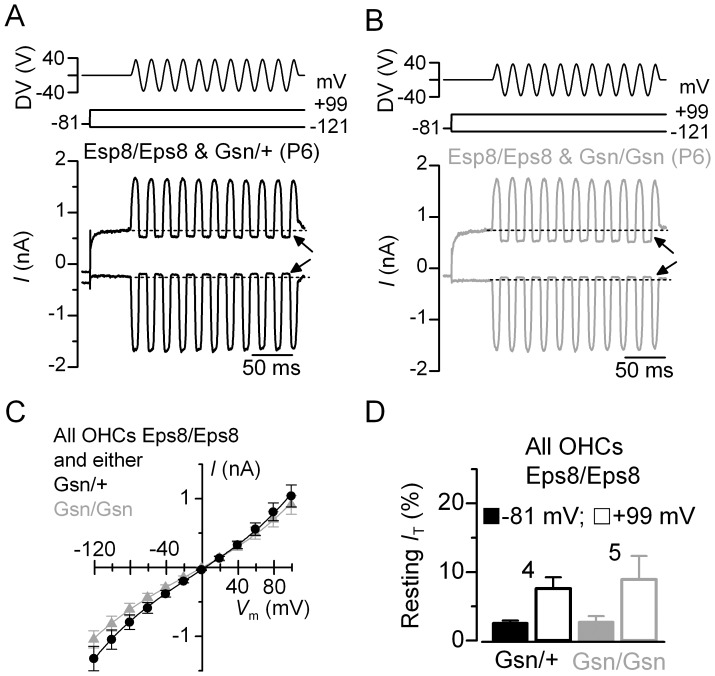
Mechanotransducer currents in OHCs from Eps8-gelsolin double mutant mice (*Eps8^tm1Ppdf^/+; Gsn^tm1Djk^/+*). A and **B**, Saturating MET currents recorded from apical-coil OHCs of mice that were all knockout for Eps8 (*Eps8/Eps8*) and either heterozygous (*Gsn/+*: **A**, black lines) or homozygous (*Gsn/Gsn*: **B**, grey lines) for the gelsolin mutant allele. MET currents were elicited by applying sinusoidal force stimuli as described in **Figure 1**. The arrows indicate the resting MET current at hyperpolarized and depolarized membrane potentials. Dashed lines indicate the holding current. **C**, Peak-to-peak current-voltage curves were obtained from four *Eps8/Eps8*; *Gsn/+* mutants and five *Eps8/Eps8*; *Gsn/Gsn* mutants (P6). The fits through the data are according to eqn.1 (see Methods) with values: *Eps8/Eps8*; *Gsn/+k* = 372±23, *V*_r_ = 3.0±0.2 mV, *V*_s_ = 43 ±2 mV, and γ = 0.47±0.01; *Eps8/Eps8*; *Gsn/Gsn k* = 321±20, *V*_r_ = 0.1±0.3 mV, *V*_s_ = 45±2 mV, and γ = 0.46±0.01. **D**, Size of the resting transducer current measured at −81 mV and +99 mV in *Eps8/Eps8*; *Gsn/+* and *Eps8/Eps8*; *Gsn/Gsn* OHCs.

We proceeded to analyse MET currents in Eps8-gelsolin double homozygous comparing responses to the double heterozygous at the gelsolin locus. The results further support the evidence that gelsolin is not required for MET in immature OHCs (**Figure 6A-C**). The main abnormality in the MET currents, when both proteins were absent, was the reduced resting current at hyperpolarized and depolarized membrane potentials (**Figure 6D**) when compared to that observed in gelsolin knockout mice (**Figure 1D**). However, the smaller resting current is comparable to that observed when only Eps8 was absent (**Figure 6A**; see also [**12**]).

## Discussion

We have shown that the actin-binding protein gelsolin is not involved in mechano electrical transduction in OHCs from pre-hearing mice. We have also shown that the role of gelsolin does not overlap with Eps8, which is another actin-binding protein expressed in the stereocilia of OHCs. While Eps8 is crucial for the initial growth of the stereociliary bundles in both IHCs and OHCs [**11]**, **[12]**, gelsolin is likely to be mainly involved in the maintenance of the hair bundle of apical coil OHCs in the mature cochlea (see also [**10**]).

### Gelsolin is not directly required for the mechano-electrical transducer current

Gelsolin is expressed in the stereocilia of apical coil OHCs from about P0 to P15 [**10**]. In the absence of gelsolin, stereocilia appear to grow normally but by the onset of hearing (P12) stereocilia in the apical turn become long and straggly, indicating its involvement in actin regulation [**10**]. We found that normal transducer currents could be elicited in immature OHCs in the absence of gelsolin, suggesting that this stereociliary protein is not essential for mechano-electrical transduction in developing OHCs, as previously shown in the absence of other hair bundle proteins such as Eps8L2 [**13**] and myosin XVa [**26**]. However, the disorganized hair bundles present in post-hearing OHCs of gelsolin knockout mice [**10**] could impact on the relation between force applied to the bundles and their movement (i.e. hair bundles would have a reduced stiffness).

### Distribution of gelsolin and Eps8 proteins in the hair cell stereociliary bundle

In mammalian cochlear hair cells, Eps8 and Eps8L2 are expressed at the tips of the stereocilia of immature and adult OHCs and IHCs [**12]**, **[13]**, while gelsolin is only transiently present along the stereocilia shaft of OHCs between P0 and P15 [**10**]. The correct translocation of both Eps8 and gelsolin, but not Eps8L2, within the stereocilia has been shown to require the motor protein MyoXVa and the scaffolding protein whirlin [**10]**, [11], **[13]**. Despite the overlapping distribution and trafficking mechanism, we found that the locations of Eps8 and gelsolin are independent of each other, suggesting a non-overlapping function in OHCs.

### Gelsolin and Eps8 have distinct but complementary roles in stereocilia formation

The staircase structure mainly develops postnatally when stereociliary elongation stops initially in the shortest rows at around postnatal day 5 (P5) and the tallest row at about P15 in mice [**6]**, **[27]**. While the growth of this fine structure requires the continuous turnover of actin filaments that form the core of each stereocilium [**28]**, **[29]**, its maintenance in mature stereocilia seems to require a much slower turnover, possibly to ensure that once established it is maintained through adult life [**30**]. Several stereociliary proteins are known to contribute to the normal development and/or maintenance of hair bundle structure and function [**6]**, **[31]**. Among these proteins, Eps8 and twinfilin 2 are directly involved in stereocilia growth since when absent (Eps8 [**12**]) or overexpressed (twinfilin 2 [**27**]) they prevent the normal development of theirstaircase architecture in both OHCs and IHCs. Although Eps8 and Eps8L2 are structurally complementary [**32**], Eps8L2 is essential for the long-term maintenance of hair bundles in fully mature OHCs and IHCs [**13**]. In the absence of gelsolin the initial growth of stereocilia occurs normally, but towards the final stage of development they become long and straggly in the apical turn [**10**], most likely due to the loss of cell-surface specializations or links that connect adjacent stereocilia [**33**]. Gelsolin is part of the whirlin complex that includes a number of proteins such as p55 and 4.1R [**25**], which are implicated in actin polymerization and cytoskeletal reorganization. However, it is possible that whirlin may scaffold multiple complexes, including a Myo15-whirlin-Eps8 complex involved with stereocilia growth. Gelsolin could act through a separate Myo15-whirlin complex with p55 and other proteins involved in actin polymerisation to govern a distinct role. Therefore, the role of gelsolin in OHCs could be to regulate the interaction between other actin binding proteins and thus stabilize the hair bundle structure once it has formed.

## Materials and Methods

### Ethics Statement

In the UK, all animal studies were licensed by the Home Office under the Animals (Scientific Procedures) Act 1986 and were approved by the University of Sheffield Ethical Review Committee and the MRC Harwell Ethical Review Committee.

### Single-hair cell electrophysiology

Outer hair cells from gelsolin knockout or gelsolin-Eps8 double mutant mice and their littermate controls [**10]**, **[12]** were studied in acutely dissected cochleae at postnatal day 6 (P6), where the day of birth is P0. Cochleae were dissected in normal extracellular solution (in mM): 135 NaCl, 5.8 KCl, 1.3 CaCl_2_, 0.9 MgCl_2_, 0.7 NaH_2_PO_4_, 5.6 D-glucose, 10 Hepes-NaOH. Sodium pyruvate (2 mM), MEM amino acids solution (50X, without L-Glutamine) and MEM vitamins solution (100X) were added from concentrates (Fisher Scientific, UK). The pH was adjusted to 7.5 (osmolality ∼308 mmol kg^−1^). All experiments were performed at room temperature (22–24°C).

Voltage recordings were performed using an Optopatch (Cairn Research Ltd, UK) amplifier. Patch pipettes were coated with surf wax (Mr. Zogs SexWax, USA) to minimise the fast capacitance transient of the patch pipette. The intracellular solution of the patch pipette (2–3 MΩ) contained (in mM): 131 KCl, 3 MgCl_2_, 1 EGTA-KOH, 5 Na_2_ATP, 5 Hepes-KOH, 10 Na_2_-phosphocreatine (pH 7.3; osmolality ∼296 mmol kg^−1^). Data acquisition was controlled by pClamp software using Digidata boards (Molecular Devices, USA). Recordings were low-pass filtered at 2.5 kHz (8-pole Bessel), sampled at 5 kHz and stored on computer for off-line analysis (Origin: OriginLab, USA). Membrane potentials in voltage clamp were corrected for the voltage drop across the uncompensated residual series resistance (*R*_s_: 1.2±0.2 MΩ, *n* = 10, after up to 80% compensation) and for a liquid junction potential (–4 mV).

Mechano-electrical transducer (MET) currents were elicited by stimulating the hair bundles of OHCs using a fluid jet from a pipette (tip diameter 8–10 µm) driven by a piezoelectric disc [**17]**, **[18]**. The pipette tip of the fluid jet was positioned near to the bundles to elicit a maximal MET current. Mechanical stimuli were applied as force-steps or 50 Hz sinusoids (filtered at 0.25 kHz, 8-pole Bessel) with driving voltages of±40 V. MET currents were recorded with a patch pipette solution containing (in mM): 106 Cs-glutamate, 20 CsCl, 3 MgCl_2_, 1 EGTA-CsOH, 5 Na_2_ATP, 0.3 Na_2_GTP, 5 Hepes-CsOH, 10 sodium phosphocreatine (pH 7.3). Membrane potentials were corrected for the liquid junction potential (–11 mV).The peak MET current- voltage curves (**Figure 1C**) were fitted according to a simple single-energy-barrier model [**34**]: *I*(*V*) = *k* [exp ((1 −γ)(*V*−*V*_r_ )/*V*_s_ ) − exp (−γ(*V*−*V*_r_ )/*V*_s_)] (eqn.1), where *k* is a proportionality constant, *V*_r_ is the reversal potential, *V*_s_ is a measure for the steepness of the rectification, and γ is the fractional distance within the membrane's electrical field of an energy barrier, as measured from the outside.

### FM1-43 Labelling

FM1-43 experiments were performed on acutely dissected apical coils of control and knockout cochleae at P10-P11 from gelsolin mice. Cochleae were bath treated with a solution containing 3 µM FM1-43 for 10 seconds, and immediately washed several times with normal extracellular solution. Images were taken within 15 minutes after exposure to FM1-43 using a CCD camera (SPOT-JNR). The cochleae were then viewed with an upright microscope equipped with epifluorescence optics and FITC filters (excitation 488 nm, emission 520 nm) using a 63X water immersion objective. Stock solutions of 3 mM FM1-43 were prepared in water. A total number of 6 control and knockout cochleae from 3 mice were used. These experiments were performed at room temperature (22–25°C) as previously described [**21**].

### Statistical analysis

Statistical comparisons of means were made by Student's two-tailed *t*-test. Mean values are quoted ± s.e.m. where *P*<0.05 indicates statistical significance.

### Antibodies and Immunohistochemistry

Goat anti-gelsolin (N-18) was purchased from Santa Cruz Biotechnology, Inc. Eps8 monoclonal antibody (610143) was purchased from BD Biosciences. Whole-mount immunostaining of the mouse cochlea with the anti-gelsolin antibody was performed as described [**35**] while that for immunostaining with anti-Eps8 was performed as described [**11**].

### Scanning Electron Microscopy

Freshly dissected inner ears were fixed for 3 to 4 hours in 2.5% gluteraldehyde in 0.1 M phosphate buffer pH 7.3 at 4°C. After 3 washes of 15 minutes in 0.1 M phosphate buffer, the ears were decalcified in 4.3% EDTA in 0.1 M phosphate buffer pH 7.3 for 48 hours after which they were dissected to expose the organ of Corti. The ears were then dehydrated in ethanol, critical point dried, sputter coated with gold and viewed on a JEOL 6010LA Scanning Electron microscope.
